# Improving the Ablation Properties of Liquid Silicone Rubber Composites by Incorporating Hexaphenoxycyclotriphosphonitrile

**DOI:** 10.3390/nano13030563

**Published:** 2023-01-30

**Authors:** Hao Zhang, Jinfeng Tian, Liwei Yan, Shengtai Zhou, Mei Liang, Huawei Zou

**Affiliations:** The State Key Laboratory of Polymer Materials Engineering, Polymer Research Institute, Sichuan University, Chengdu 610065, China

**Keywords:** ablative materials, liquid silicone rubber, thermal insulation, ablation performance

## Abstract

The ablative properties of epoxy-modified vinyl silicone rubber (EMVSR) composites containing hexaphenoxycyclotriphosphonitrile (HPCTP) have been systematically studied. The strength of the ablation char layer was greatly enhanced with the addition of HPCTP, which induced the formation of a more complete, denser, and thicker char during oxyacetylene ablation tests. Moreover, the HPCTP-containing EMVSR composites demonstrated lower thermal conductivity and pyrolysis rate when compared with those without HPTCP. At the same time, the thermal insulation properties of HPCTP-filled composites were improved under low heat flow ablation scenarios. The reduction of graphitic carbon content, the formation of phosphate-like crystals as well as the increase of SiC content contributed to strengthening the char layer, which was critical for improving the ablation properties. The optimum char layer strength and thermal insulation properties were achieved when the content of HPCTP was 15 phr, whereas an optimum ablation resistance was achieved at 25 phr HPCTP. This suggests that HPCTP-modified EMVSR composites can be used for thermal protection purposes, especially in the fields of aerospace and aeronautics.

## 1. Introduction

A thermal protection system (TPS) is key to ensuring the normal service and operation of space vehicles. Ablative materials play a key role in the TPS [1,2,3,4], which are mainly used for protecting the surface and propulsion systems [5,6,7,8,9]. Rigid ablative materials have poor deformability and thermal insulation performance, making it difficult to meet the requirements of complex sealing structures on space vehicles [10], while flexible ablative materials outperform rigid ablative materials in terms of sealing, deformation adaptability, and thermal insulation performance [11,12].

Conventionally, flexible ablative materials are prepared using nitrile rubber (NBR) [13], EPDM rubber [14], silicone rubber [15], and polyphosphazene [16] as the host matrices. Among these materials, silicone rubber is an organic-inorganic elastomeric polymer that exhibits the advantages of high elasticity, corrosion resistance, and non-toxicity. In addition, the formation of the ceramic-like protective layer in silicone rubber composites that consists of the silica (SiO_2_) complex during ablation helps to resist the erosion caused by oxygen flow into the substrate, thereby enhancing oxidation resistance and heat insulation properties. However, pure silicone rubber shows poor compatibility with inorganic fillers and a poor carbonization rate, which leads to poor ablative performance and, therefore, it cannot be directly used for TPS purposes. As previously reported [17], epoxy-modified vinyl silicone rubber (EMVSR) as a matrix showed better ablation properties when compared with its unmodified counterpart. A comparison of the properties such as shear strength, tensile strength, elongation at break, and char residual at 800 °C (i.e., R_800_) between the EMVSR and unmodified liquid silicone rubber are listed in Table 1. It is clear that the overall performance of EMVSR outperforms the unmodified counterpart. Therefore, EMVSR was selected as the matrix for the present study.

Enhancing the char formation ability and improving the scouring resistance of the char layer are believed to be critical for improving the ablation resistance of TPS. A pure polymer matrix cannot form a high-quality char layer to withstand the erosion of the heat sources. Therefore, inorganic fillers are normally added to improve the quality of the char layer, thereby enhancing the ablative performance. However, the addition of a large number of inorganic fillers impairs the flexibility and processability of the ablative composites. Moreover, the thermal conductivity and density are increased as well, which are not favorable for their practical application as flexible ablative composites [18,19,20,21]. Hexaphenoxycyclotriphosphonitrile (HPCTP), which contains a molecular skeleton structure with alternating P and N atoms and multiple external aromatic units, has been widely employed to improve the flame retardancy of silicone rubber [22] and many other polymers [23,24,25]. To the best of the author’s knowledge, very few studies have been conducted to improve the ablative properties of silicone rubber-based materials by incorporating the HPCTP.

In the present work, the influence of HPCTP addition on the ablative properties of EMVSR-based composites was evaluated. The ablative properties, pyrolysis, and ablation mechanisms for HPCTP-containing EMVSR composites have been explored in depth. A facile strategy for the preparation of high-performance ablation-resistant, thermal insulative flexible ablative materials that can be potentially applied in the fields of aerospace and aeronautics etc., has been identified. This work may have great significance for expanding the application of EMVSR in TPS.

## 2. Experimental Sections

### 2.1. Materials

Epoxy-modified vinyl silicone rubber (EMVSR) was prepared as previously described [17]. Briefly, an equivalent amount of epoxy resin (E44) and silane coupling agent (KH570) were mixed and heated to 130 °C with extensive external stirring under inert gas atmosphere. Afterward, a defined amount of catalyst tetraisopropyl titanate was added. The reaction was continued until the mixture became a homogeneous viscous liquid. The crude product was thoroughly washed with methanol to remove the KH570. Then, the obtained product was termed EMVSR, and it was used as the matrix. Fumed silica (SiO_2_) was purchased from Degussa (Frankfurt, Germany). Short carbon fibers (CF) with a length of 3 mm were purchased from Toho (Tokyo, Japan). The curing agent, hydrogen-containing silicone oil (HS, S-H content 0.35 mol/100 g), was supplied by Guangzhou Tianling Company (Guangzhou, China). Hexachloroplatinic acid (HCPA) was supplied by China BlueStar Chengrand Co., Ltd. (Chengdu, China). Hexaphenoxycyclotriphosphonitrile (HPCTP) was purchased from Shanghai Kunzhen Materials Company (Shanghai, China). All products were used as received.

### 2.2. Sample Preparation

**Figure 1:**
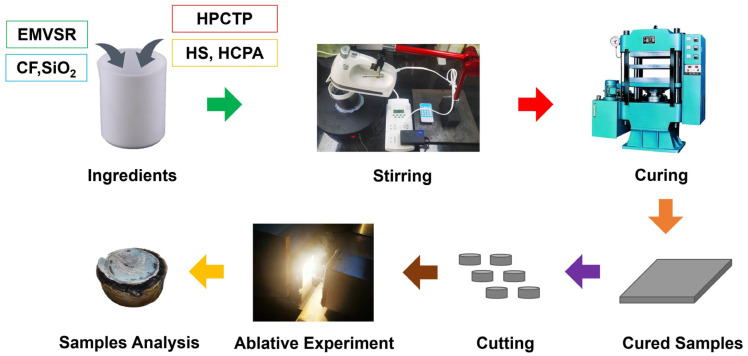
Schematic illustration of preparing HPCTP-containing EMVSR composites and subsequent ablation tests.

Firstly, EMVSR, SiO_2_, CF, HPCTP, HS, and HCPA were charged in a ceramic beaker, and they were extensively stirred by an automatic stirring device. Subsequently, the composites were cured in a plate vulcanizer at 80 °C and 10 MPa for 2 h. Then, the cured samples were cut into standard ablation specimens (Φ30 × 10 mm^3^) for subsequent oxyacetylene ablation tests. The formulations of the studied systems are tabulated in Table 2, and the sample preparation process is depicted in Figure 1. The samples were named as per the content of HPCTP in the composites. For example, HPCTP-0 indicates that the content of HPCTP in EMVSR-based composites was 0, while HPTCP-5 refers to the samples containing 5 phr HPCTP. 

### 2.3. Characterization

#### 2.3.1. Oxyacetylene Ablation Tests

The ablation tests were conducted using an oxyacetylene ablative machine (ZR-323A, Xi’an Zhirui Co., Ltd., Xi’an, China). The pressure of acetylene (C_2_H_2_) and oxygen (O_2_) were 0.095 and 0.4 MPa, respectively. The samples with a dimension of Φ30 × 10 mm^3^ were tested under a heat flux of approximately 4 MW/m^2^. The ablative test for each specimen lasted 30 s, and five replicates were tested. The height and weight of the samples before and after ablation tests were measured. Then, the ablated char was removed from the ablated samples, and the remaining height was recorded. The line ablation rate (R*_l_*), mass ablation rate (R*_m_*), pyrolysis rate (R*_p_*), and carbonization rate (R*_c_*) were calculated as per the following equations [26]:

R*_l_* = (d_1_ − d_2_)/t (1)

R*_m_* = (m_1_ − m_2_)/t (2)

R*_p_* = (d_1_ − d_3_)/t (3)

R*_c_* = (d_2_ − d_3_)/t (4)

where, d_1_ is the height of the samples before the ablation tests, d_2_ is the height after ablation tests, d_3_ is the height after removing the char layer, t is the ablation time (i.e., 30 s), m_1_ is the weight of the samples before ablation and m_2_ represent the weight after ablation tests.

Samples that were treated under a lower heat flux (i.e., 0.2 MW/m^2^) were tested using the butane ablation test, as described elsewhere [27].

#### 2.3.2. Other Characterizations

The thermal conductivity was measured using a Hot disk thermal constant analyzer (TPS 2500, Sweden). The thermal stability was evaluated using a thermogravimetric analyzer (TGA, TG209F1 Iris, Netzsch, Germany) under nitrogen (N_2_) atmosphere. The compression strength of the char layer was measured by an electronic universal testing machine (UTM4204, Shenzhen Suns Technology Stock Co., Ltd., China), as described elsewhere [28]. Density was used to quantify the compactness of char layer, which was measured using an electronic densimeter (DH-300, DahoMeter, China) [29]. The surface and cross-sectional microstructure of the char layer was observed using a field emission scanning electron microscope (SEM, Apreo S HiVoc, the United States) equipped with energy-dispersive spectroscopy (EDS). X-ray diffraction (XRD) analysis was performed using a Philips X-ray diffractometer (DY1291, Holland) to analyze the phase composition of the ablated char. The in-line back-face temperature of ablative samples was recorded using a K-type thermocouple thermometer. Raman spectroscopy was employed to study the quality of the char layer [30]. The Thermogravimetric-Fourier Transform Infrared Spectra-Gas Chromatography-Mass Spectroscopy (TG-FTIR-GCMS) tests were performed according to the literature [31]. Briefly, the samples were heated from room temperature to 800 °C at a ramping rate of 50 °C/min under N_2_, and FTIR spectrum ranged from 400 to 4000 cm^−1^ was simultaneously recorded. The ablation surface temperature was monitored using a dual colorimetric online infrared thermometer (SA-2S300A,1000–3000 °C, Wuxi Shiao Soft Technology Co., Ltd., Wuxi, China).

## 3. Results and Discussion 

### 3.1. Thermogravimetric Analysis (TGA)

**Figure 2:**
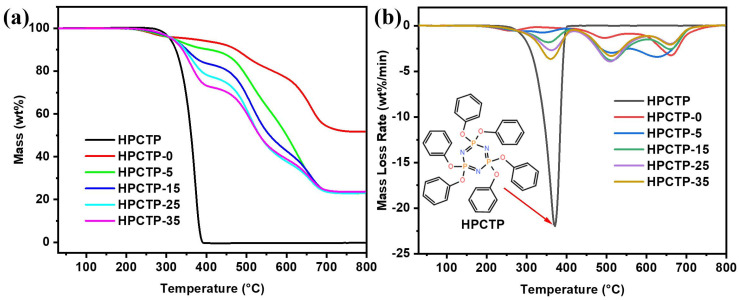
(**a**) TG and (**b**) DTG curves of HPTCP and HPCTP-containing EMVSR composites.

The TGA and derivative TG (DTG) curves for HPCTP-containing EMVSR composites are given in Figure 2a and Figure 2b, respectively. The typical thermal analysis data, including initial thermal degradation temperature (T_5%_), maximum degradation temperature (T_max_), and the char residue at 800 °C (R_800_), are tabulated in Table 3. Figure 2a shows that the decomposition of HPCTP mainly occurs between 336 and 385 °C, and the char residue of HPTCP is close to zero after 385 °C, suggesting that HPCTP is fully decomposed under heating treatment. The associated reaction is expressed as follows:

HPCTP→CO_X_↑+N_X_Oy↑+H_2_O↑+Volatile phosphorus-containing compounds↑

In addition, Figure 2a shows that the thermal stability of EMVSR-based composites degrades significantly with the addition of HPTCP. For example, the T_5%_ of HPCTP-0 is 366 °C, and it is reduced to 319 °C with the addition of 5 phr HPTCP. Table 3 indicates that there is no obvious correlation between the values of T_5%_ and the content of HPTCP, suggesting that the low onset decomposition temperature of HPCTP is believed to be the contributing factor that leads to the deterioration of thermal stability for HPCTP-containing composites. The R_800_ of HPCTP-0 is 51.72%, indicating the excellent char-formation ability of EMVSR. However, the R_800_ of HPTCP-filled EMVSR composites drops to approximately 23%, irrespective of the HPTCP-filling content. This suggests that the thermal stability of EMVSR-based composites deteriorates with the presence of HPTCP. However, the values of T_max1_ gradually shift towards higher temperature regions with the increasing addition of HPTCP. T_max1_ represents the breakage of epoxy chains in EMVSR and the dehydration of unreacted hydroxyl groups in epoxy, indicating that the HPCTP delays the breakage of epoxy molecules and dehydration of unreacted hydroxyl groups, but it also causes a gradual increase of the decomposition rate. T_max2_, which is related to the rearrangement of silicone rubber macromolecular chains. Similar to T_max1_, the T_max2_ moves towards the high-temperature regions, but it also increases the maximum degradation rate with the presence of HPCTP. In this scenario, the water generated during the pyrolysis of HPCTP promotes the degradation of the molecular chains of silicone rubber. T_max3_ is related to the carbonization of the benzene ring into carbon residue, and the filling of HPCTP decreases the maximum degradation rate of T_max3_, indicating that HPCTP inhibits the degradation of the benzene ring into carbon.

### 3.2. Thermal Conductivity and Heat Absorption Properties

**Figure 3:**
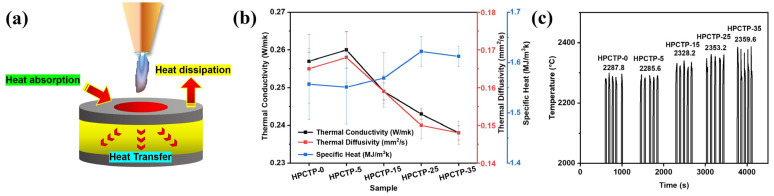
HPCTP-modified EMVSR composites: (**a**) schematic diagram of heat loss, (**b**) thermal conductivity, thermal diffusivity, and specific heat curves, (**c**) surface temperature curve of ablated samples.

Figure 3a is a schematic illustration of heat losses involved in the ablation process. The loss of heat mainly consists of heat absorption, heat dissipation, and heat transfer. Heat absorption includes thermal degradation and heat capacity endothermic, and heat dissipation is mainly caused by thermal radiation and mechanical exfoliation of char fragments at high temperatures [4,32].

Figure 3b exhibits the thermal conductivity and thermal diffusivity curves as well as the specific heat curves of HPCTP-containing EMVSR composites, respectively. It can be seen that the thermal conductivity and thermal diffusivity of EMVSR-based composites experiences an initial increasing and then decreasing trend with the increasing addition of HPCTP. Lowing the thermal conductivity is an important trend in the research of ablation materials [33]. Therefore, the introduction of HPCTP provides a strategy for the preparation of flexible ablation-resistant materials with low thermal conductivities.

On the whole, the ability of the material’s heat capacity is enhanced with the increase of HPCTP, but its increasing rate is lower than the decreasing rate of thermal conductivity and thermal diffusivity. The reason is that the intrinsic thermal degradation of HPCTP absorbs more heat, and this change becomes more obvious with the increasing addition of HPCTP. Figure 3c shows the surface temperature change curves of HPCTP-loaded EMVSR composites during ablation measurements. Five replicates were tested for each sample, and the reported value is an average of the maximum surface temperature for the tested specimens. In general, the surface temperature increases with the addition of HPCTP at higher loading concentrations. Given the same testing setup, the surface temperature of the tested materials is determined by the heat loss during the ablation process. It can be seen that the surface temperature is negatively correlated with thermal conductivity, implying that lower thermal conductivity favors the accumulation of heat in the central ablated region. The increase of HPCTP content leads to a reduction of thermal conductivity, which in turn hinders the transfer of heat into the sample block. The accumulation of heat in the central region thus leads to an increase in the surface temperature. Although the heat absorption capacity is slightly enhanced, the increasing rate of specific heat is smaller than that of thermal conductivity. As a result, heat conduction plays a more significant role in determining the surface temperature of the ablated samples.

### 3.3. Ablation Properties

**Figure 4:**
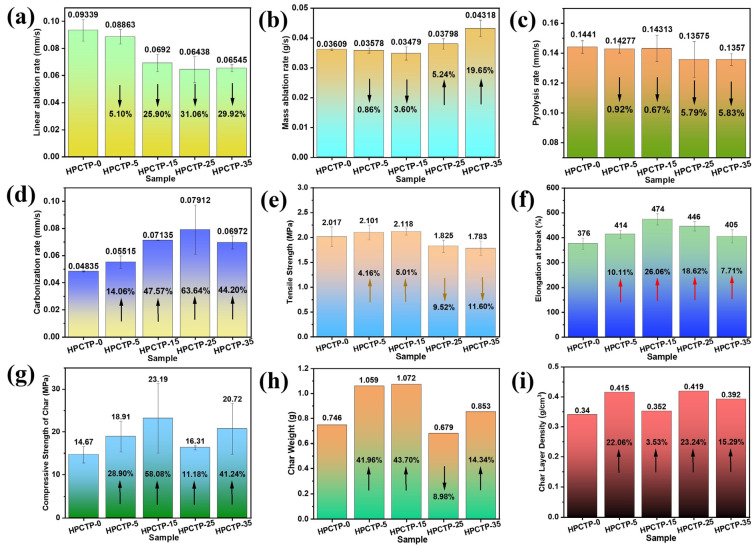
The ablation properties of HPCTP-containing EMVSR composites: (**a**) line ablation rate, (**b**) mass ablation rate, (**c**) pyrolysis rate, (**d**) carbonization rate, (**e**) tensile strength and (**f**) elongation at break of the samples before ablation tests, (**g**) compressive strength, (**h**) weight and (**i**) density of the char layer.

The ablation and mechanical performance of HPCTP-containing EMVSR samples are displayed in Figure 4. The line ablation rate (R*_l_*) is considered as one of the most important indicators that characterize the ablation performance of ablative materials. The values of R*_l_* for HPCTP-containing EMVSR composites are exhibited in Figure 4a. Results indicate that the R*_l_* is reduced with the introduction of HPCTP. In particular, the R*_l_* of EMVSR-based composite with 25 phr HPCTP reaches 0.064 mm/s, which is about 31.1% lower than the unfilled EMVSR counterpart. This is related to the reduction of thermal conductivity upon adding HPCTP, which greatly lowers the erosion of external heat. The increase in specific heat is regarded as another influencing factor [34]. More importantly, the presence of HPCTP promotes the formation of a strong char layer (as reported in the supporting information), which protects the internal matrix from being eroded by oxyacetylene flame. In addition, the formed gases during the thermal degradation of HPCTP could take away the heat, thereby lowering the further erosion of the internal matrix. Moreover, HPCTP contains P and N elements which play a significant role in inhibiting combustion and reducing thermal erosion. Thus, the above factors lead to a reduction in the R*_l_* of HPCTP-containing EMVSR composites.

The mass ablation rate (R*_m_*) of HPCTP-filled EMVSR composites is given in Figure 4b. Results suggest that the R*_m_* is slightly lowered at low HPCTP content, whereas it increases at higher adding content (≥25 phr). Higher surface temperature normally leads to a higher R*_m_*. As displayed in Figure 3c, the surface temperature shows an initial decreasing and then increasing trend with an increase of the HPCTP content, which corresponds with the changes in R*_m_*.

Figure 4c shows that the pyrolysis rate (R*_p_*) is gradually reduced with the increasing addition of HPCTP. When the content of HPCTP was 25 phr, the R*_p_* reached as low as 0.136 mm/s, which is 5.62% lower than that of HPCTP-0. Although the R*_p_* of HPCTP-35 is slightly lower than HPCTP-25, the optimum content of HPCTP is 25 phr by considering the processability, cost, and many other factors that incurred by adding 35 phr HPCTP.

The carbonization rate (R*_c_*) indicates the formation speed of the char layer and the thickness of the char layer after the ablation process, which has a direct relation to the ablation and erosion resistance of ablative composites. Generally speaking, the thicker the char layer, the less influence of the thermal oxygen damage or erosion on the matrix beneath the char layer. Figure 4d shows that the R*_c_* increases with the addition of HPCTP, indicating that adding HPCTP is beneficial to increase the thickness and the formation rate of the char layer. The R*_c_* is 0.079 mm/s when the content of HPCTP is 25 phr, which is 63.64% higher than HPCTP-0. The reason for the formation of a thicker char layer is that the pyrolysis of HPCTP generates a large amount of volatile gas, which expands the char layer. In addition, HPCTP generates benzene-like compounds, which are more likely deposited in the inner char layer [35], thereby thickening the char layer.

The tensile strength and elongation at the break of HPCTP-containing EMVSR composites are given in Figure 4e,f. The tensile strength and elongation at the break of the material have a direct effect on the erosion resistance of ablative materials, which in turn affects the ablation and thermal insulation properties [7,36]. As shown in Figure 4e, when the content of HPCTP is 15 phr, the EMVSR-based composite has the highest tensile strength (i.e., 5.01% higher than HPCTP-0) and elongation at break (26.06% higher than HPCTP-0). Compared with HPCTP-0, the elongation at break of EMVSR-based composites increases to the highest at 15 phr HPCTP, and it decreases with further increasing HPCTP content when the content of HPCTP is 25 phr, the elongation at the break increases by 18.62% when compared with HPCTP-0, which is consistent with the excellent ablation performance of HPCTP-25.

The compressive strength, weight, and density of the char layer are displayed in Figure 4g, h, and i, respectively. The results indicate that the compressive strength and weight of the char layer show a positive correlation. Both the compressive strength and weight of the char layer increase with the addition of HPCTP, and they reach the highest at 15 phr HPCTP. Under high temperatures, HPCTP reacts with EMVSR to form complex P-containing and N-containing silicides, which is advantageous to improve the compactness and strength of the char layer [see Figure 4g], thereby enhancing the anti-ablation performance. The compressive strength of the char layer of HPCTP-containing composites is higher than HPCTP-0, which is consistent with the images of the peeled char layer and the largest char fragment, as displayed in Figure S1. The compressive strength of the char layer for HPCTP-15 is 58.1% higher than HPCTP-0, which is likely related to the higher R*_p_* of EMVSR with 15 phr HPCTP. Figure 4i shows that the density of the char layer lies in a range of 0.34–0.42 g/cm^3^, and the density of the char layer of HPCTP-containing samples is higher than the pure EMVSR, corresponding to the improvement of the compactness of char layer. The density of the char layer for HPTCP-25 increases by 23.2% when compared with HPTCP-0, which is favorable for resisting the erosion of heat flow, thereby improving the ablation resistance. Thus, the addition of HPCTP enhances the strength [see Figure 4g] and the density of the char layer [see Figure 4i], which is favorable for reducing the R*_p_* during the ablation testing process.

### 3.4. Thermal Insulation

**Figure 5:**
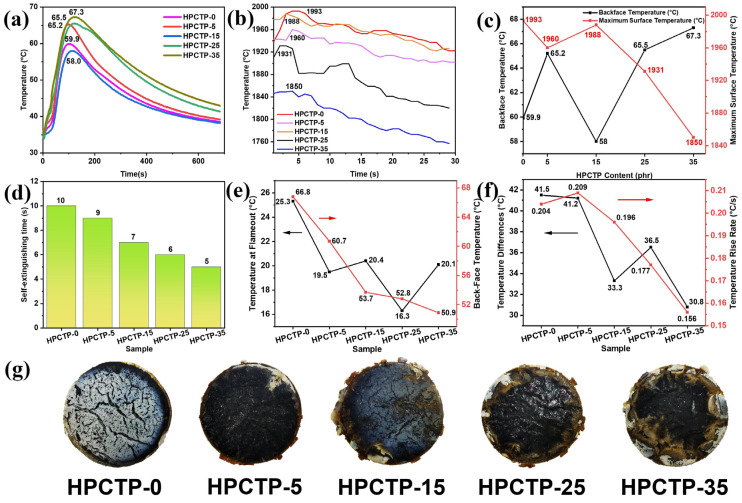
Thermal insulation-related performance parameters of HPCTP-containing EMVSR composites: (**a**) back-face temperature curve under 4 MW/m^2^ heat flow; (**b**) surface temperature curve recorded during back-face temperature measurement under 4 MW/m^2^ heat flow; (**c**) the relationship between maximum back-face temperature and maximum surface temperature; (**d**) self-extinguishing time of the flame on the surface of sample after ceasing oxyacetylene ablation experiments; (**e**) ceasefire temperature and maximum back-face temperature under 0.2 MW/m^2^ heat flow; (**f**) back-face temperature difference and back-face temperature rise rate under 0.2 MW/m^2^ heat flow; (**g**) optical images of the samples after butane ablation tests.

Figure 5a–d present the thermal insulation properties of the EMVSR-based material under 4 MW/m^2^ heat flow. The lower the maximum back-face temperature, the better the thermal insulation performance. Figure 5a shows that the maximum back-face temperature tends to increase with an increase in HPCTP content, but the thermal insulation performance does not significantly deteriorate. The back-face temperature of HPCTP-25, which has the best ablation performance, is only 5.6 °C higher than HPCTP-0. When the HPCTP is 15 phr, the maximum back-face temperature is 1.9 °C lower than that of HPCTP-0, and the thermal insulation performance is improved by 3.17%, indicating that the presence of HPCTP improves the thermal insulation properties of EMVSR. The main reason for the lower back-face temperature is that HPCTP-15 has the best mechanical strength and char layer strength (see Figure 4), which is beneficial to resist heat flow erosion, thus improving thermal insulation performance.

Figure 5b shows that the overall surface temperature gradually decreases with an increase in HPCTP content. To explore the reason for the change in surface temperature, the relationship between maximum back-face temperature and maximum surface temperature is established, as exhibited in Figure 5c. Figure 5c shows that a negative correlation is observed between both factors. This is because surface temperature is closely related to heat transfer. When the back-face temperature is higher, the heat input through the ablated surface penetrates more inside the material and to the back-face, which contributes to a decrease in the surface temperature. The trend of back-face temperature is consistent with the density of the char layer [Figure 4i], and a slight difference is detected in HPCTP-35 because the back-face temperature is also affected by the line ablation rate. The line ablation rate of HPCTP-35 is higher than HPCTP-25, which means that the distance from the ablated surface to the back-face is smaller for HPCTP-35, and the shortening of heat transfer distance increases the back-face temperature. The higher the density of the char layer, the denser the char layer. The thermal conductivity of the char layer skeleton is much higher than air in the pores. Therefore, a denser char layer tends to have higher thermal conductivity, thereby resulting in a higher back-face temperature.

Figure 5d displays the self-extinguishing time curve of samples when the oxyacetylene ablator is stopped. With the increase of HPCTP content, the self-extinguishing time is gradually reduced, indicating that the addition of HPCTP is beneficial in reducing the flammability of EMVSR composites. The flames on the surface of silicone rubber after ablation are caused by the release of combustible gases such as hydrogen, carbon monoxide, and methane during the pyrolysis of the matrix. The reduced self-extinguishing time for HPCTP-containing EMVSR composites indicates that the release of combustible gases and the associated combustion reaction is inhibited. Therefore, the introduction of HPCTP facilitates the inhibition of the release of combustible gases from the substrate and the combustion reactions. In addition, HPTCP plays a role as a flame retardant since it contains P and N, which is favorable for inhibiting the degradation and thermal erosion of the internal matrix during the ablation process, thus improving the ablation resistance of EMVSR-based composites.

Figure 5e–g provide the relevant characterization of thermal insulation properties of EMVSR-based composites under a 0.2 MW/m^2^ heat flow using a butane flame. The temperature at flameout was determined at the moment of ablation cessation. The temperature difference is the difference between the maximum back-face temperature and instantaneous back-face temperature at a cease-fire, which indicates the ability of the material to continue to rise in back-face temperature after ceasing ablation tests. The temperature difference is divided by the temperature rising time to obtain the temperature rise rate. Figure 5e indicates that the HPCTP could effectively improve the thermal insulation performance of EMVSR-based composites. Compared with HPCTP-0, the maximum back-face temperatures of HPCTP-25 and HPCTP-35 decrease by 20.96% and 23.80%, respectively.

Figure 5f shows that the temperature difference decreases with increasing content of HPCTP, indicating that a large amount of HPCTP is beneficial to suppress the temperature increase inside the material. In addition, the temperature rise rate shows a trend of rising and then falling, which is consistent with the trend of thermal conductivity [Figure 3b]. The butane ablation test shows that HPCTP reduces the cease-fire temperature and maximum back-face temperature, the temperature difference, and the temperature rise rate, which improve the thermal insulation performance at low heat flows. This is because the dosing of HPCTP strengthens the char layer, improves the denseness of the char layer, increases the thickness of the char layer, and causes a decrease in thermal conductivity. These factors could improve the ablation resistance and resist the erosion of the substrate, leading to the improvement of thermal insulation performance for EMVSR-based composites.

Figure 5g demonstrates that many large cracks are formed on the surface of HPCTP-0. These large cracks allow heat to penetrate the internal matrix, leading to an increase in back-face temperature. On the surface of the HPCTP-containing samples, a denser char layer is formed with fewer large cracks, which is consistent with the improved thermal insulation properties. Thus, the presence of HPCTP improves the thermal insulation of EMVSR at low heat flow and ensures that the thermal insulation performance does not significantly deteriorate at high heat flows.

### 3.5. Morphology of the Char Layer

**Figure 6:**
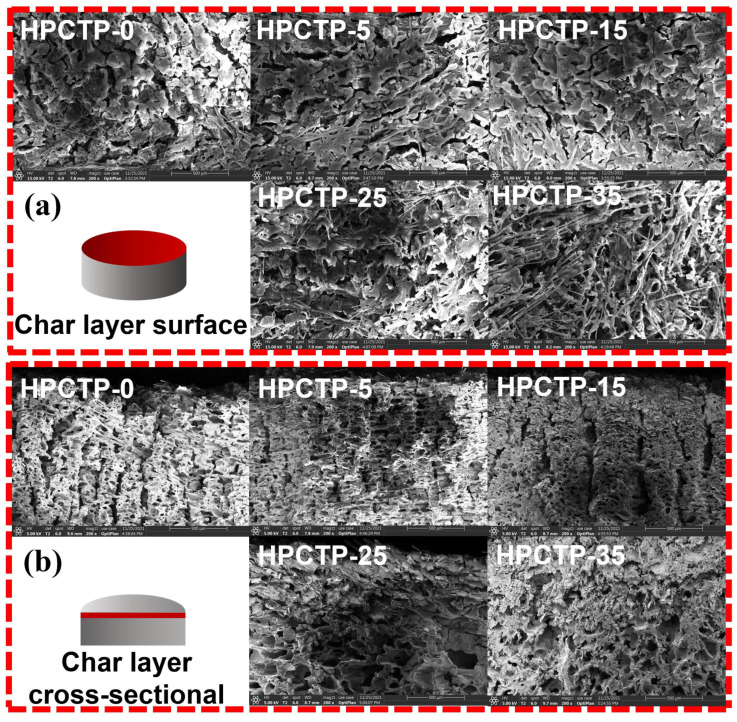
Morphology of the char layer of HPCTP-containing EMVSR composites under 4 MW/m^2^ heat flow: (**a**) the surface and (**b**) cross-sectional morphology of char layer.

The microstructure of the char layer for HPCTP-containing EMVSR composites is displayed in Figure 6a. The results indicate that the surface of the HPCTP-0 char layer is relatively loose and demonstrates many large-size cracks, thereby resulting in a decrease in the ablation performance. After filling HPCTP, the fraction of large-size cracks in the char layer gradually decreases, indicating that the quality of the char layer is improved. The improvement of the quality of the char layer surface corresponds to the improvement of ablation performance, which indicates that the presence of HPCTP could reduce or inhibit the formation of large-size cracks and improve the denseness of the char layer, which helps to improve the strength of the char layer.

Figure 6b gives the cross-sectional microstructure of the char layer for HPCTP-containing EMVSR composites. The results show that many interconnected large and long straight channels appear in HPCTP-0, which lead to a significant deterioration of the ablation performance. After filling HPCTP, the number of long channels in the char layer of HPCTP-containing composites is reduced, and smaller pores and closed porous structures are produced, which is consistent with the improvement of the compactness of the char layer and ablative performance of HPCTP-containing EMVSR composites.

**Figure 7:**
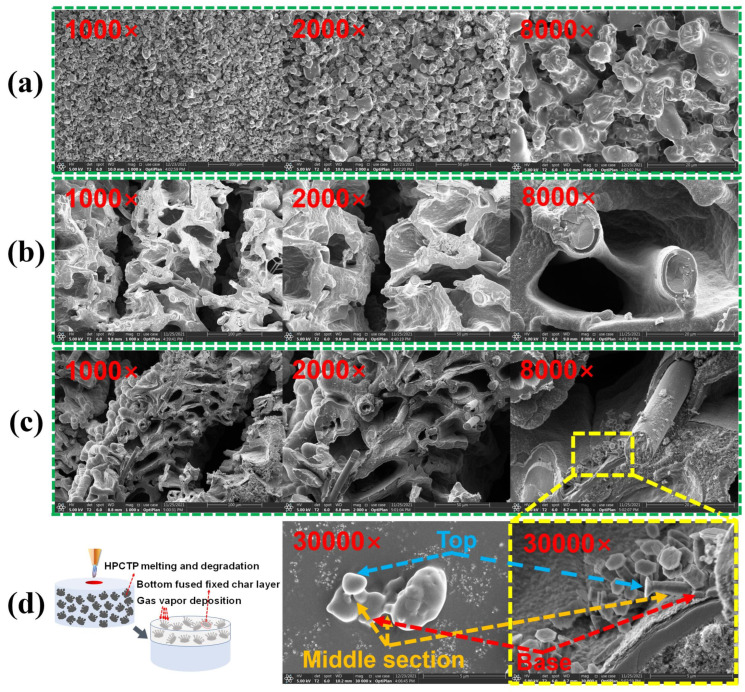
Morphology of the char layer at different magnifications: (**a**) HPCTP; (**b**) HPTCP-0; (**c**) HPTCP-15; (**d**) schematic diagram of the formation of phosphate crystal structure.

To explore the reason for the HPCTP-enhanced char layer, the microstructures of the char layer of HPCTP-0 and HPCTP-15 are compared, as exhibited in Figure 7. Figure 7a shows the morphology of intrinsic HPCTP, while Figure 7b and Figure 7c show the morphology of the char layer at different magnifications for HPCTP-0 and HPCTP-15, respectively. The char layer of HPCTP-15 is much denser and contains prismatic crystals. However, the prismatic crystals of HPCTP are not visible at different magnifications, excluding other raw materials such as silicone rubber matrix, silica, and CF in microscopic form. Therefore, the prismatic crystals are formed during the ablation process, which is advantageous to improve the ablative properties of EMVSR composites.

Figure 7d shows the prismatic crystal formation mechanism and the microstructure of HPCTP at 30,000× magnification and the prismatic crystal morphology at 30,000× magnification, respectively. HPCTP intrinsically has an irregular prismatic crystal structure with a large base and a small head, and the formation of prismatic crystals after ablation is consistent with vapor phase deposition and pyrolysis. Therefore, the formation of prismatic crystals is related to the melting and pyrolysis of HPCTP under a high-temperature environment [36]. The base of the prismatic crystals is tightly bound to the char layer, which could effectively bond and fix the local char layer and act as a reinforcement of the char layer, thus improving the ablative properties of EMVSR-based composites.

### 3.6. Composition of the Ablated Composite

**Figure 8:**
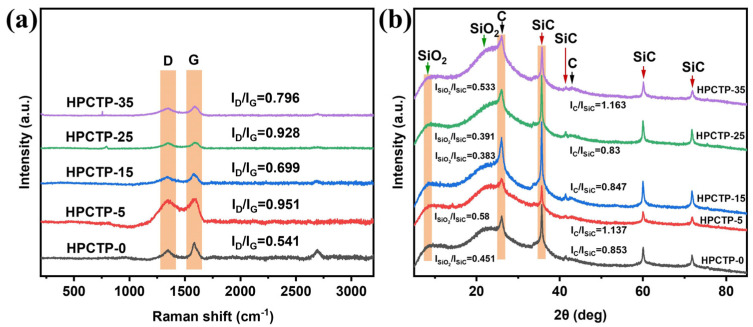
Char layer of HPCTP-containing EMVSR composites after ablation tests: (**a**) Raman spectra; (**b**) XRD curves.

Figure 8a displays the Raman spectra of the char layer for HPCTP-containing EMVSR composites. The peak that originated from about 1350 cm^−1^ is related to the disordered structure in the char layer (i.e., D band), whereas the peak that was detected in the vicinity of 1600 cm^−1^ is designated as the G band, which is related to the existence of crystalline structure. The quality of the char layer is characterized by ratios of D band to G band, namely I_D_/I_G_, which is calculated from the peak heights of both the D and G peaks [37]. Graphitic carbon crystals have a laminar structure with weak interlayer bonding; therefore, the strength of the char layer is reduced with increasing graphitic carbon content. Thus, it is desirable to reduce the graphitization of the char layer to facilitate the improvement of the strength of the char layer [38]. The increase in the I_D_/I_G_ ratio of the material after filling HPCTP indicates that the graphitization of carbon is reduced, which is beneficial to reduce the generation of laminated graphitic carbon and increase the interlayer bonding of carbon, which is consistent with the increase of the strength of the char layer.

Figure 8b shows the XRD patterns of the char layer and the curves derived from the ratio of peak heights of silica, carbon, and silicon carbide (SiC). It is clear that the main components of the char layer are SiO_2_, C, and SiC, and no significant difference is observed among different samples. This is because many intolerant substances escape as volatile gases during ablation tests. Figure 8b indicates that the ratios of I(SiO_2_)/I(SiC) and I(C)/I(SiC) for both HPCTP-15 and HPCTP-25 are lower than HPCTP-0. This indicates that the content of SiC in the char layer of HPCTP-15 and HPCTP-25 increases. SiC has excellent high-temperature resistance and oxidation resistance. Therefore, the increase in the fraction of SiC improves the oxidation and high-temperature resistance of the char layer and thus helps improve the char layer’s strength.

**Figure 9:**
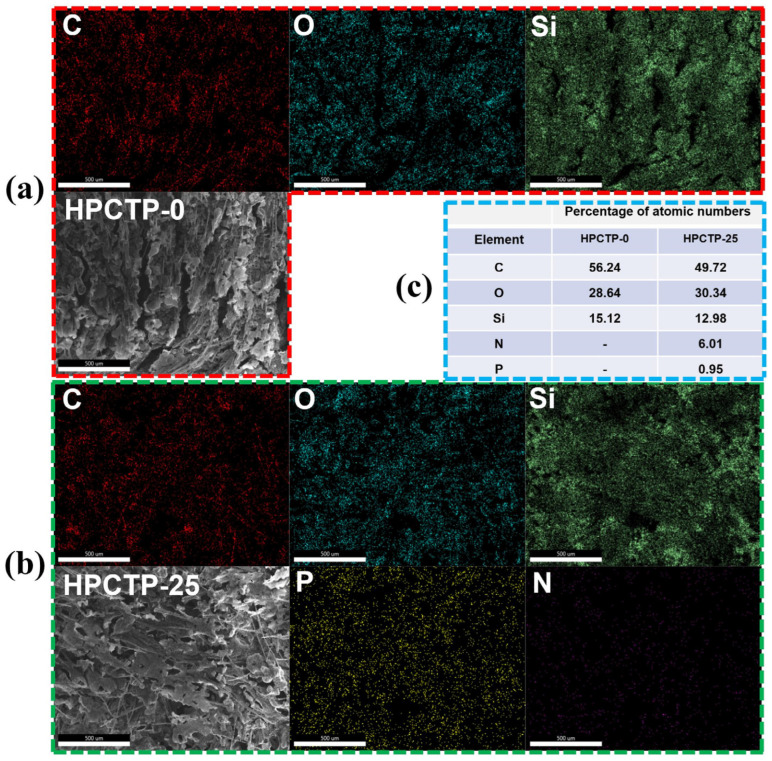
Elemental analysis of the char layer of the sample after ablation: Mapping images on the surface of char layer of (**a**) HPCTP-0; (**b**) HPCTP-25; (**c**) the relative content of elements related to the EDS on the surface of the char layer.

Figure 9a–c shows the mapping images of the char surface and EDS images of the char surface for HPCTP-0 and HPCTP-25, respectively. The char surface of HPCTP-25 is composed of C, O, Si, N, and P. It indicates that HPCTP is involved in the ablation reaction on the surface. The pyrolysis products of HPCTP would have a complex high-temperature reaction with EMVSR on the surface of the char layer, generating solid residues containing N and P that remain on the surface of the char layer. The uniform distribution of P and N indicates that the solid residues containing P and N are uniformly distributed on the surface of the char layer, which helps to improve the denseness and strengthen the char layer. The distribution of P on the surface of the char layer also justifies the presence of phosphate crystals in Figure 8. Figure 9c shows that the molar content of N is higher than that of P, but in the molecular structure of HPCTP, the molar content of N and P is at the same ratio. The above results indicate that the compounds containing N have better heat resistance than those containing P, and the substances containing P on the surface of char layer escape pyrolytically in an ultra-high-temperature environment.

### 3.7. Analysis of Thermal Degradation Mechanism

**Figure 10:**
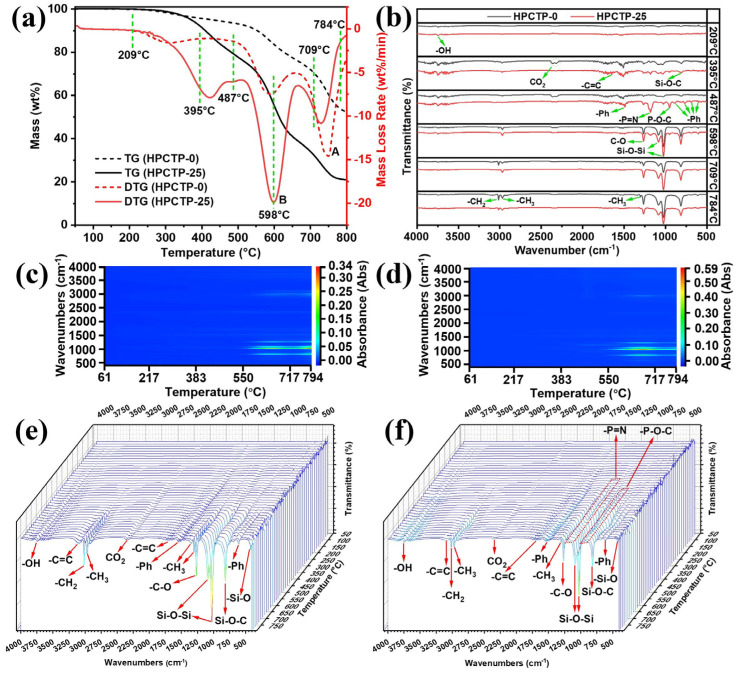
TG-FTIR-GCMS tests of HPCTP-0 and HPCTP-25 under nitrogen atmosphere: (**a**) TG-DTG curves of HPCTP-0 and HPCTP-25; (**b**) infrared spectra of gases that released from HPCTP-0 and HPCTP-25 at different temperatures; distribution of volatile gases at different temperatures: (**c**) HPCTP-0; (**d**) HPCTP-25; three-dimensional infrared spectra of the released gases: (**e**) HPCTP-0, (**f**) HPCTP-25.

**Figure 11:**
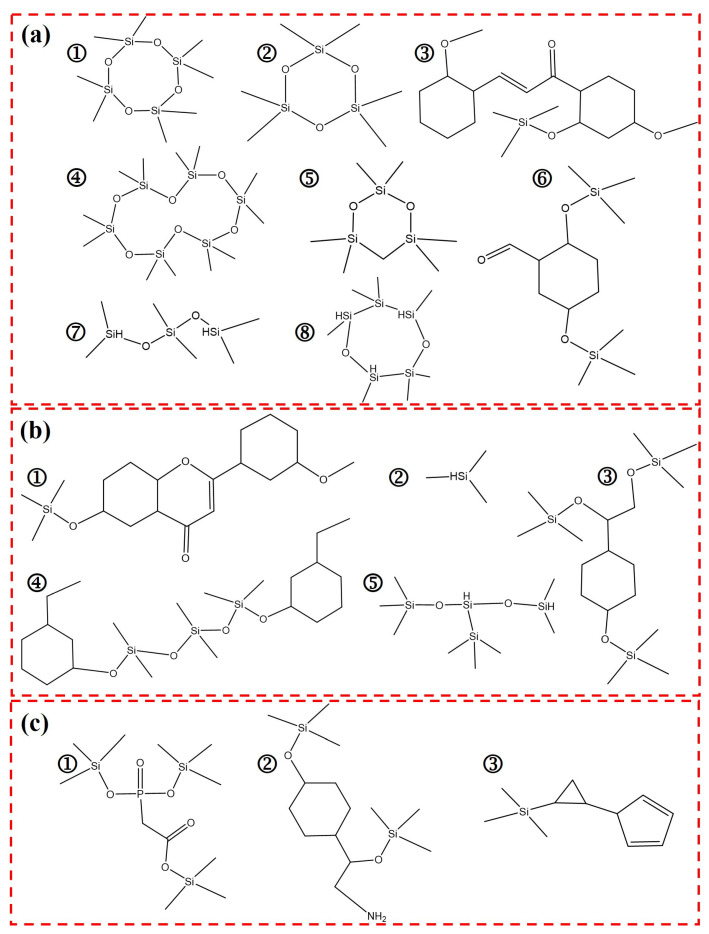
The molecular structures of the gaseous products released at the peaks of respective DTG maximum degradation rates for HPCTP-0 and HPCTP-25: (**a**) pyrolysis products in both HPCTP-0 and HPCTP-25; (**b**) pyrolysis products only appear in HPCTP-0; (**c**) pyrolysis products only appear in HPCTP-25.

To analyze the thermal degradation mechanism of HPCTP-containing EMVSR samples, HPCTP-0 and HPCTP-25 were tested by TG-FTIR-GCMS. Figure 10a displays the TG-DTG curves of HPCTP-0 and HPCTP-25. Some characteristic temperatures in the selected DTG curves are linked with the FTIR information to obtain the infrared spectra of the volatile gases released by HPCTP-0 and HPCTP-25 at different temperatures [Figure 10b]. Figure 10c,d are the distribution of volatile gases at different temperatures for HPCTP-0 and HPCTP-25, respectively. Figure 10e,f are the three-dimensional infrared spectra of the gases released by HPCTP-0 and HPCTP-25, respectively. Table 4 shows the relationship between the infrared spectra and the corresponding groups in the TG-FTIR-GCMS tests. Figure 11 is the molecular formula of the released gas products at the maximum degradation peak of DTG for HPCTP-0 [peak A in Figure 10a] and the peak of the maximum degradation rate of DTG for HPCTP-25 [peak B in Figure 10a]. These molecular formulae were analyzed by GCMS assay. Figure 11a is the gas product produced by pyrolysis contained in both HPCTP-0 and HPCTP-25. Figure 11b is the gaseous product of pyrolytic evolution contained only in HPCTP-0. Figure 11c is the pyrogenic gas release product contained only in HPCTP-25.

Figure 10a shows that the degradation rate of the B peak of HPCTP-25 increases compared with the second peak of DTG for HPCTP-0, which indicates that the pyrolysis of HPCTP promotes the degradation of EMVSR. Figure 10c,d show that the pyrolysis gas release mainly occurs after 383 °C, with wavenumbers around 400–1500 and 3000 cm^−1^. This is consistent with the curve changes in Figure 10e,f. In Figure 10b, there are fewer peaks at low temperatures, while more peaks appear at high temperatures. Table 4 shows that at 209 °C, the released gas is mainly water. Peaks of -CH_3_, -C=C, Si-O-C, and -OH are detected at 395 °C, representing the release of water, CO_2_, and benzene ring-containing compounds to form cyclosiloxane gaseous substances which correspond to the cleavage of epoxy resin molecular chain and the initial pyrolysis of silicone rubber. At 395, 487, and 598 °C, HPCTP-25 shows distinct peaks of -P=N, P-O-C, and benzene rings (these peaks were not present in HPCTP-0). This is related to the intrinsic pyrolysis of HPCTP after 395 °C, leading to the removal of P-, N-containing compounds, and benzene ring structures. At 598 °C, peaks of C-O, Si-O-Si, -CH_2_, -CH_3_, and Si-O-C are clearly present. This indicates that at this temperature, it mainly corresponds to the cleavage of intrinsic molecular chains of silicone rubber in EMVSR, resulting in the formation of cyclosiloxane-like small molecules. The peaks at 709 and 784 °C are consistent with those at 598 °C. It can be seen that there is a weak -C=C peak at 3100 cm^−1^. It indicates that at higher temperatures, i.e., 709 and 784 °C, it is mainly the breakage of the molecular chain of silicone rubber and the removal of a benzenoid ring-like structure. In addition, the peaks of -P=N and P-O-C gradually weaken at 709 and 784 °C, indicating that the pyrolysis of HPCTP is complete before 709 °C.

Compared with HPCTP-0, HPCTP-25 exhibits -P=N and -P-O-C peaks and more benzene ring peaks, which is consistent with the structure of HPCTP. When HPCTP is ablated and pyrolyzed in EMVSR-based composites, a complex pyrolysis reaction occurs. Due to the blockage of the char layer, vapor phase deposition occurs in the inner char layer away from the ablated surface, and the gases containing N, P, and benzene rings react with the groups on the molecular structure of EMVSR, which contributes to the denseness of the char layer.

Figure 11a depicts the molecular structure of gaseous products released by HPCTP-0 at 750 °C [peak A in Figure 10a] and HPCTP-25 at 600 °C [peak B in Figure 10a]. It shows that the two largest pyrolysis peaks of EMVSR, which correspond to T_max2_ and T_max3_, are mainly from the scission of the molecular chain of silicone rubber, resulting in a large number of complex cyclic siloxanes and linear siloxanes. Figure 11b illustrates that EMVSR at a higher temperature of 750 °C also produces a variety of aromatic siloxane and linear siloxane structures. In addition, small molecules are detected [Figure 11b], which result from the pyrolytic cleavage of the end groups on EMVSR, and the released large cyclic siloxane end groups. Figure 11c shows that HPCTP-25 generates P-containing siloxane compounds, N-containing siloxanes, and complex cyclosiloxane compounds in addition to the gaseous products in Figure 11a at 600 °C, which is consistent with the previous mapping and EDS results. This indicates that HPCTP reacts with EMVSR during pyrolysis and generates gases that contain P-Si and N-Si compounds. Under oxyacetylene testing conditions, these gases undergo vapor deposition between the surface of the char layer and the original matrix, and thus a dense char layer is constructed. In addition, the molecular formula of the phosphorus-silicon compound [Figure 11c] has a structure similar to that of phosphate, which provides a reasonable explanation for the formation of phosphate-like crystals that strengthen the char layer [39,40,41,42], thereby improving the anti-scouring and anti-ablation properties of EMVSR-based composites.

**Figure 12:**
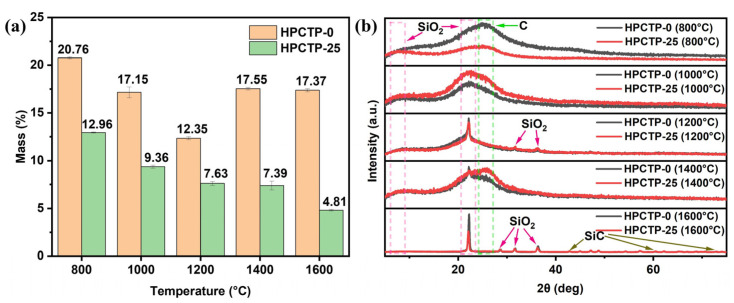
Static ablation of HPCTP-0 and HPCTP-25 at different temperatures in a tube furnace: (**a**) residual mass percentage; (**b**) XRD patterns of carbon residue.

Figure 12 gives the XRD patterns of the residual carbon and residual mass of HPCTP-0 and HPCTP-25 in the tube furnace from 800 to 1600 °C. In Figure 12a, the residual mass of HPCTP-25 gradually decreases with increasing temperatures, and the residual mass of HPCTP-25 is lower than that of HPCTP-0 at the same temperatures, indicating that the HPCTP-filled system releases more gaseous products during the pyrolysis process, but at the same time, the release of a large amount of P-, N-containing and benzene rings provides more vapor deposition on the char layer, thus improving the ablation resistance of corresponding composites.

In Figure 12b, it can be seen that at 800 °C, the main components of residual carbon consist of SiO_2_ and C. Among them, HPCTP-0 has more ordered C than HPCTP-25, which corresponds to the higher graphitization of HPCTP-0, as confirmed by the Raman spectral analysis, and also corresponds to the lower strength of the char layer of HPCTP-0. At 1000 °C, the residual carbon is mainly composed of SiO_2_ and disordered carbon. At 1200 °C, the residual carbon is also mainly composed of SiO_2_ and disordered carbon, but the crystalline form of SiO_2_ is more ordered, and the peak is sharper when compared with that observed at 1000 °C. At 1400 °C, it can be seen that the main components of the residual carbon of both HPCTP-0 and HPCTP-25 are SiO_2_ and disordered C. The SiO_2_ peak produced by HPCTP-0 is sharper than HPCTP-25, and the C peak that is produced by HPCTP-0 is slightly sharper than HPCTP-25. In contrast, the C peak of HPCTP-25 is more subdued, which is consistent with the previous Raman results, indicating that HPCTP-25 produces more disordered C, corresponding to the higher strength of the char layer. Surprisingly, the peak of SiC appears at 1600 °C, which coincides with the appearance of SiC peaks in the char layer of the samples after ablation tests, as displayed in Figure 8b. The formation of SiC improves the high-temperature resistance and oxidation resistance of the char layer and thus provides some strength, which contributes to improving the ablation performance of the HPCTP-containing EMVSR composites. In addition, the peaks observed at 1600 °C are sharper when compared with those observed at 800~1400 °C, indicating more ordered crystalline forms of derivatives are formed in the residual carbon. It can thus be expected that more complex pyrolytic carbonization occurs during the oxyacetylene ablation tests within the material when compared with the tube furnace measurements.

## 4. Conclusions

In this study, the efficacy of using HPCTP to improve the ablative performance of EMVSR was evaluated. The strength and compactness of the char layer are enhanced in terms of HPTCP-containing composites, which is related to the improvement of thermal insulation performance, especially under low thermal ablation conditions. In addition, the results show that the maximum decrease in line ablation rate is 31.06% with the addition of 25 phr HPCTP, and the maximum increase of compressive strength of the char layer is 58.08% by filling 15 phr HPCTP. The pyrolysis products of HPCTP could react with EMVSR to yield P- and N-containing silicides, which contributes to strengthening and densifying the char layer. Moreover, the generation of less graphitic carbon and the presence of phosphate-like crystal structures in the char layer is believed crucial to improving the ablation resistance of HPCTP-containing EMVSR composites. Furthermore, more SiC is generated in the char layer of the HPCTP-containing system under higher temperatures, which contributes to improving the quality of the char layer and ablation resistance. Thus, the above results indicate that the addition of HPCTP significantly improves the ablation resistance of EMVSR composites, which demonstrates promising applications in the aerospace industry.

## Figures and Tables

**Table 1:** The properties of unmodified silicone rubber and EMVSR [17].

Sample	Unmodified Liquid Silicone Rubber	EMVSR
Shear strength (MPa)	0.31	0.81
Tensile strength (MPa)	0.32	0.83
Elongation at break (%)	95.8	240.5
R_800_ (%)	51.22	53.53

**Table 2:** The Formulation of HPCTP Modified EMVSR-Based Composites.

Sample	EMVSR (phr)	SiO_2_ (phr)	CF (phr)	HPCTP (phr)	HS (phr)	HCPA (phr)
HPCTP-0	100	10	6	0	3	0.2
HPCTP-5	100	10	6	5	3	0.2
HPCTP-15	100	10	6	15	3	0.2
HPCTP-25	100	10	6	25	3	0.2
HPCTP-35	100	10	6	35	3	0.2

phr: parts per hundred silicone rubber by weight.

**Table 3:** Thermal analysis parameters of HPTCP and HPCTP-containing EMVSR composites.

Sample	T_5%_ (°C)	R_800_ (%)	T_max1_ (°C)	T_max2_ (°C)	T_max3_ (°C)
HPCTP-0	366	51.72	261	496	661
HPCTP-5	319	23.00	339	514	629
HPCTP-15	318	23.44	353	513	658
HPCTP-25	319	23.05	364	509	659
HPCTP-35	316	23.67	361	511	659

**Table 4:** Relationship between infrared spectrum and corresponding group in TG-FTIR-GCMS test.

Wavenumber (cm^−1^)	Corresponding Chemical Group
3609–3852	-OH
3016	-CH2 (Naphthenic)
2968, 1304	-CH_3_
2365, 2322	CO_2_
3100, 1650	-C=C
1268	C-O (Aryl ether)
1181	-P=N
1086, 1024	Si-O-Si
952	-P-O-C
1512, 886, 768, 690, 606	-Ph
814	Si-O-C

## Supplementary Materials

The following supporting information can be downloaded at: https://www.mdpi.com/article/10.3390/nano13030563/s1, Figure S1: Images of HPCTP-containing EMVSR composites after ablation tests: (a) top view, (b) side view, (c) char layer, (d) the largest char fragment. Ref. [43] is cited in Supplementary Materials.

## References

[1] Uyanna O., Najafi H. (2020). Thermal protection systems for space vehicles: A review on technology development, current challenges and future prospects. Acta Astronaut..

[2] Kumar C.V., Kandasubramanian B. (2019). Advances in Ablative Composites of Carbon Based Materials: A Review. Ind. Eng. Chem. Res..

[3] Gudivada G., Kandasubramanian B. (2019). Zirconium-doped hybrid composite systems for ultrahigh-temperature oxidation appli-cations: A review. Ind. Eng. Chem. Res..

[4] Natali M., Kenny J.M., Torre L. (2016). Science and technology of polymeric ablative materials for thermal protection systems and propulsion devices: A review. Prog. Mater. Sci..

[5] Sziroczak D., Smith H. (2016). A review of design issues specific to hypersonic flight vehicles. Prog. Aerosp. Sci..

[6] Fahy W.P., Langston J., Wu H., Koo J.H., Kim S., Misasi D., Parra R., Canan L., Li K. (2020). Silica–Phenolic Nanocomposite Ablatives for Thermal Protection Application. J. Spacecr. Rocket..

[7] Bocchio J.A., Escobar M.M., Amado J.C.Q. (2020). Ablative Properties of Polyurethanes Reinforced with Organoclay. Polym. Eng. Sci..

[8] Hou Y., Yee C., Li W., Koo J.H., Li L., Rech B., Fahy W., Wu H., Buffy J.J. (2022). A novel ablative material for thermal protection system: Carbon fiber/polysiloxane composites. Aerosp. Sci. Technol..

[9] AJ A.J., Panigrahi S.K., Sasikumar P., Rao K.S., Krishnakumar G. (2022). Ablative properties, thermal stability, and compressive behaviour of hybrid silica phenolic ablative composites. Polym. Degrad. Stab..

[10] Arabgol F., Kokabi M., Bahramian A.R. (2018). Ablation behavior of organoclay-NBR insulator: Modeling and experimental. Fire Mater..

[11] George K., Panda B.P., Mohanty S., Nayak S.K. (2018). Recent developments in elastomeric heat shielding materials for solid rocket motor casing application for future perspective. Polym. Adv. Technol..

[12] Ji Y., Han S., Xia L., Li C., Wu H., Guo S., Yan N., Li H., Luan T. (2021). Synergetic effect of aramid fiber and carbon fiber to enhance ablative resistance of EPDM-based insulators via constructing high-strength char layer. Compos. Sci. Technol..

[13] Amado J.C.Q., Ross P.G., Sanches N.B., Pinto J.R.A., Dutra J.C.N. (2020). Evaluation of elastomeric heat shielding materi als as insulators for solid propellant rocket motors: A short review. Open Chem..

[14] Qu H., Hui K., Bian C., Guan Y., Li H., Luan T., Yan Y. (2022). Lightweight and Mechanically Robust EPDM Foams for High Thermal Insulation and Moderate Ablative Resistance Via Constructing Cellular Char Layer. Macromol. Mater. Eng..

[15] Liu Z., Huang Y., Yan L., Chen Y., Liang M., Zou H. (2022). Mesophase pitch modified silicone rubber coatings with fence-like ceramic layer structures and excellent ablation resistance performance. Prog. Org. Coatings.

[16] Zhang S., Wang J., Zhu H., Ali S., Ma H., Zhang L., Wu D., Wu Z. (2017). Evaluation of poly (diaryloxyphosphazene) elastomer for heat shielding insulations and morphology of charred layers. High Perform. Polymers.

[17] Wang Y., Zhang B., Zhou S., Chen Y., Liang M., Zou H. (2020). Preparation of high-performance epoxy-containing silicone rubber via hydrosilylation reaction. J. Appl. Polym. Sci..

[18] Zhang G., Wang F., Huang Z., Dai J., Shi M. (2016). Improved ablation resistance of silicone rubber composites by introducing mont morillonite and silicon carbide whisker. Materials.

[19] Guan Y., Zhang L., Zhang L., Lu L. (2011). Study on ablative properties and mechanisms of hydrogenated nitrile butadiene rubber (HNBR) composites containing different fillers. Polym. Degrad. Stabil..

[20] Yang D., Zhang W., Jiang B., Guo Y. (2013). Silicone rubber ablative composites improved with zirconium carbide or zirconia. Compos. Part A Appl. Sci. Manuf..

[21] Liu Y., Duan H., Huang Q. (2022). Multiscale effect of graphene oxide with short carbon fiber for property improvement of room temperature vulcanized silicone rubber. Polym. Bull..

[22] Pang Q., Deng J., Kang F., Shao S. (2020). Effect of expandable Graphite/Hexaphenoxycyclotriphosphazene beads on the flame retard ancy of silicone rubber foam. Mater. Res. Express..

[23] Shen L., Li J., Lin H., Feng S., Zhang Y. (2017). The enhanced compatibility and flame retarding ability of UHMWPE-MH compo-sites by adding phenoxycyclophosphazene (HPCTP). Polym. Bull..

[24] Wang X., Li Q., Di Y., Xing G. (2012). Preparation and properties of flame-retardant viscose fiber containing phosphazene deriva-tive. Fiber. Polym..

[25] Shen L., Shao C., Li R., Xu Y., Li J., Lin H. (2019). Preparation and characterization of ethylene–vinyl acetate copolymer (EVA)–magnesium hydroxide (MH)–hexaphenoxycyclotriphosphazene (HPCTP) composite flame-retardant materials. Polym. Bull..

[26] Gao G., Zhang Z., Zheng Y., Jin Z. (2010). Effect of fiber orientation angle on thermal degradation and ablative properties of short-fiber reinforced EPDM/NBR rubber composites. Polym. Compos..

[27] Cai Y., Yan L., Wang Y., Ge Y., Liang M., Chen Y., Zou H., Zhou S. (2022). A room temperature self-healing and thermally re-processable cross-linked elastomer with unprecedented mechanical properties for ablation-resistant applications. Chem. Eng. J..

[28] Liu Z., Chen Z., Yan L., Liang M., Zou H. (2021). Ordered graphitized ceramic layer induced by liquid crystal epoxy resin in silicone rubber composites with enhanced ablation resistance performance. Mater. Chem. Phys..

[29] Li S., Zhang H., Yan L., Zhou C., Heng Z., Liang M., Zou H., Chen Y. (2022). The effect of layered materials on the ablation resistance and heat insulation performance of liquid silicone rubber. Polym. Adv. Technol..

[30] Huang Y., Zhang H., Zhang X., Yan L., Ling Y., Zou H., Chen Y., Liang M. (2022). Effect of Mesophase Pitch Incorporation on the Ablation Behavior and Mechanism of Phenolic Composites. Ind. Eng. Chem. Res..

[31] Liu Z., Asghar A., Hou C., Ali I., Naqvi S.R., Wang N., Zhu H., Mehmood M.A., Liu C.-G. (2022). Co-pyrolysis of the Chinese liquor industry waste and bamboo waste, elucidation of the pyrolysis reaction chemistry, and TG-FTIR-MS based study of the evolved gases. Fuel.

[32] Lachaud J., Cozmuta I., Mansour N.N. (2010). Multiscale approach to ablation modeling of phenolic impregnated carbon ablators. J. Spacecraft. Rocket..

[33] Hou Y., Yee C., Li W., Koo J.H., Li L., Rech B., Fahy W., Wu H., Buffy J.J. Performance of a Carbon Fiber/Polysiloxane Composite: Thermal, Ablation, Flammability, and Mechanical Characterization. Proceedings of the 2022 AIAA SciTech Forum.

[34] Kucharczyk W., Dusiński D., Żurowski W., Gumiński R. (2018). Effect of composition on ablative properties of epoxy composites modified with expanded perlite. Compos. Struct..

[35] Kun H., Li J., Li K., Yan N., Bian C., Guan Y., Yang Y., Li H. (2022). Effects of temperature and pressure on chemical vapour deposition in micro-nano porous structure in char layer of polymer composites. Polym. Degrad. Stab..

[36] Eroğlu M.S. (1998). Characterization of the network structure of hydroxyl terminated poly(butadiene) elastomers prepared by different reactive systems. J. Appl. Polym. Sci..

[37] Sun M., Wang X., Ye Z., Chen X., Xue Y., Yang G. (2022). Highly Thermal Conductive Graphite Films Derived from the Graphiti zation of Chemically Imidized Polyimide Films. Nanomaterials.

[38] Xu J., Guo L., Wang H., Li W., Liu N., Wang T. (2022). Influence of graphitization temperature on microstructure and mechanical property of C/C-SiC composites with highly textured pyrolytic carbon. J. Eur. Ceram. Soc..

[39] Poojary D., Borade R., Clearfield A. (1993). Structural characterization of silicon orthophosphate. Inorg. Chim. Acta.

[40] He C., Xiao G., Jin X., Sun C., Ma P.X. (2010). Electrodeposition on Nanofibrous Polymer Scaffolds: Rapid Mineralization, Tunable Calcium Phosphate Composition and Topography. Adv. Funct. Mater..

[41] Lu X., Zhao Z., Leng Y. (2005). Calcium phosphate crystal growth under controlled atmosphere in electrochemical deposition. J. Cryst. Growth.

[42] Hui K., Wang L., Qu H., Li J., Gao C., Wang W., Bian C., Yan N., Yang Y. (2022). Thermal stability, pore structure characteristics of char layer and ablation resistance mechanism of new thermal insulation composites based on polyphosphazene polymer. Polym. Degrad. Stabil..

[43] Chen Y., Wu H., Duan R., Zhang K., Meng W., Li Y., Qu H. (2022). Graphene doped Sn flame retardant prepared by ball milling and synergistic with hexaphenoxy cyclotriphosphazene for epoxy resin. J. Mater. Res. Technol..

