# Supplementary material for: Improving the Ablation Properties of Liquid Silicone Rubber Composites by Incorporating Hexaphenoxycyclotriphosphonitrile
Source: Nanomaterials (Basel). 2023 Jan 30;13(3):563. doi: 10.3390/nano13030563 (PMC9920970; doi:10.3390/nano13030563)
Supplement: Supplementary file 1 [file nanomaterials-13-00563-s001.zip › nanomaterials-2161566-supplementary.pdf]

Supporting information

Article

Improving the Ablation Properties of Liquid Silicone Rubber Composites by Incorporating Hexaphenoxycyclotriphosphonitrile

Hao Zhang, Jinfeng Tian, Liwei Yan, Shengtai Zhou*, Mei Liang and Huawei Zou*

Correspondence: szhou@scu.edu.cn (S.Z.); hwzou@scu.edu.cn (H.Z.)

The performance of maintaining the original shape

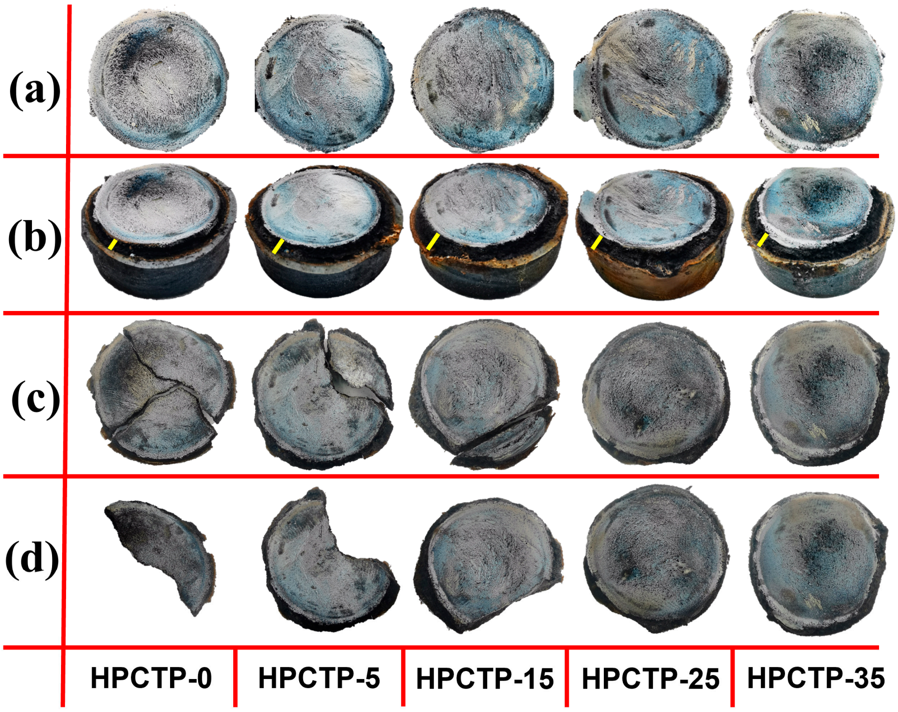

Figure S1. Images of HPCTP-containing EMVSR composites after ablation tests: (a) top view, (b) side view, (c) char layer, (d) the largest char fragment.

Figures S1(a) and S1(b) display the top and side view images of HPCTP-containing EMVSR composites after ablation tests. Figures S1(c) and S1(d) are the photos of the char layers and the largest char fragments associated with the char layer. Although the stress caused by the large amounts of gases that generated during ablation tests may cause large cracks in the top surface of the char layer, nearly all samples have intact char layers after ablation tests, as displayed in Figure S1(a). This indicates that samples could maintain their original appearance by forming a dense char layer during the ablation process. The formed char layer is intact when the concentration of HPCTP is higher than 15 phr (i.e., 25 and 35 phr), suggesting that dosing a higher content of HPTCP favors the formation of an intact dense char layer which is beneficial for the improvement of ablation performance. The above findings indicate that the rapid degradation of HPCTP might contribute to the formation of an intact char layer [1], which is crucial to improving the ablation properties of EMVSR-based composites.

The length of the yellow line that marked in Figure S1(b) indicates the distance from the unabated area to the edge of the char layer. The longer the distance, the greater the degree of expansion of char layer. It can be seen that the char layer expands with the addition of HPCTP, which is related to the gas release during the thermal degradation of HPCTP and the matrix. The expanded char layer would thus protect the internal substrate from being further ablated. Figure S1(c) shows that the char layer of HPCTP-containing samples is more complete when compared with HPCTP-0. Figure S1(d) further reveals that the size of the largest remaining char layer increases with increasing content of HPCTP, indicating that the existence of higher content of HPCTP enhances the strength of the char layer.

REFERENCE

[1] Chen, Y.; Wu, H.; Duan, R.; Zhang, K.; Meng, W.; Li, Y.; Qu, H. Graphene doped Sn flame retardant prepared by ball milling and synergistic with hexaphenoxy cyclotriphosphazene for epoxy resin. *J. Mater. Res. Technol.* **2022**, *17*, 774-788.
